# Neuroborreliosis and Post-Treatment Lyme Disease Syndrome: Focus on Children

**DOI:** 10.3390/life13040900

**Published:** 2023-03-28

**Authors:** Agnieszka Myszkowska-Torz, Magdalena Frydrychowicz, Mateusz Tomaszewski, Magdalena Figlerowicz, Anna Mania, Katarzyna Mazur-Melewska

**Affiliations:** 1 Department of Infectious Diseases and Child Neurology, Karol Marcinkowski University of Medical Sciences in Poznań, 61-701 Poznań, Poland; 2 Department of Immunology, Karol Marcinkowski University of Medical Sciences in Poznan, 61-701 Poznań, Poland; 3 Department of Orthodontics and Temporomandibular Disorders, Karol Marcinkowski University of Medical Sciences in Poznań, 61-701 Poznań, Poland

**Keywords:** neuroborreliosis, PTLSD, variable major protein-like sequence, children

## Abstract

Neuroborreliosis is a form of Lyme Borreliosis (LB) that affects various structures of the central and peripheral nervous system. Although most cases of LB can be cured with a course of antibiotics, some children can present prolonged symptoms, which may constitute post-treatment Lyme disease syndrome (PTLDS). The aim of our analysis was the long-term observation of children with NB and the determination of their risk of PTLDS. The clinical observation was supplemented by a laboratory study based on the assessment of the dynamics of anti-VlsE (variable major protein-like sequence, expressed) IgG antibodies in children with NB after antibiotic therapy. The prospective survey based on 40 children presented 1–2 forms of NB. The control group consisted of 36 patients with analogical symptoms for whom LB was excluded. Our long-term observation showed a low risk of developing long-term complications in children who received antibiotic therapy in accordance with the recommendations. The concentration of anti-VlsE IgG demonstrates a statistical significance for differences between the control and the study groups for each measurement period. Higher values of anti-VlsE IgG were observed in the study group, and the concentration decreased from the first measurement period to the next. The article emphasizes the importance of the long-term follow-up of children with neuroborreliosis.

## 1. Introduction

For many years, there has been a significant increase in the number of cases of tick-borne diseases, especially Lyme borreliosis (LB), in Europe and North America [1,2,3]. It is expected that cases will increase by another 20% in the upcoming decade, at least partially due to climate change and better tick survival during the winter [3]. Poland is the country with the highest incidence rate of LB next to Germany, Austria, Slovenia, and Sweden [4,5,6].

LB is the most widespread disease transmitted by ticks, the zoonotic source of which are small forest rodents and the vectors of ticks of the genus *Ixodes* (*I*) [7]. The etiological agent of LB is Gram-negative spirochaetes *Borrelia burgdorferi* (*Bb.*) sensu lato (*Spirochaetales*, *Spirochaetaceae*), which was first isolated in 1981 in the United States from the *I. dammini/scapularis* tick and described as a new species in 1984 [8,9]. Currently, more than 20 genospecies are described of which six are considered pathogenic for humans: *Bb.* sensu stricto, *B. garinii*, *B. afzelii*, *B. bavariensis*, *B. spielmani*, and *B. lusitaniae* [4,9,10].

Geographical distribution and clinical manifestations vary according to the species involved [11].

The infection occurs when a *Bb.*-infected tick penetrates the human skin using a suction tube. The *Borrelia* spirochetes initially spread to the site of entry, leading to a local skin infection, which untreated leads to a disseminated form. Clinically, LB may occur in two phases: an early phase, which is first localized and then disseminated, and a late phase. The early localized phase (calculated up to 8 weeks from the moment of infection) is most often associated with the occurrence of a primary lesion, i.e., a characteristic erythema migrans (EM), as a pathognomonic symptom. Another form of the early local phase is borrelial lymphocytoma, which manifests as a blue-red nodule on the earlobe, scrotum, or nipple [12,13]. In the early disseminated stage of the disease (from 6 to 26 weeks from the moment of infection), various organs are involved as a result of the dissemination of the spirochetes through the blood and lymph vessels in the body. Cardiac involvement may be associated with cardiac arrhythmias, most commonly in the form of second- and third-degree atrioventricular block. Arthritis most often affects one joint, although polyarticular manifestations are also described with the involvement of several, usually large, joints (knee, ankle, elbow, and temporomandibular joint) [14]. In the case of the nervous system, meningitis, facial nerve paralysis (FNP), or involvement of other cranial nerves and acute transverse myelitis occur [15,16,17]. In European countries, the most common form of neuroborreliosis in adults is lymphocytic meningitis (Bannwarth syndrome) [10]. This form is less common in children [6]. The early disseminated phase also includes multiple erythema migrans (MEM), where it takes on multiple forms occurring simultaneously on different parts of the body [12,13]. The late phase of LB in children occurs several months, sometimes even several years, after infection in the form of Lyme arthritis (mono-, oligo-, or polyarthritis), acrodermatitis chronica athrophicans, and neuroborreliosis (NB) [18].

In North America, LB is caused by *Bb.* sensu lato, which is characterized by joint symptoms. In Europe and Asia, at least five species of spirochetes of the genus *Borrelia* (*Bb.* sensu stricto, *B. afzelii*, *B. garinii*, *B. bissettii*, and *B. lusitaniae*) are responsible for Lyme disease, which greatly diversifies the clinical picture of the disease [10,16]. In Poland, the most common spirochetes are *Borrelia afzelii* and *Borrelia garinii*. This is related to the high incidence of EM (approximately 80%) and NB (10%–15%) in European children [4,10,19].

Although most cases of LB can be cured with a course of antibiotics, some children can present prolonged symptoms, such as pain, fatigue, or concentration difficulty, which can last more than 6 months after they finish therapy. This situation is called Post-Treatment Lyme Disease Syndrome (PTLDS) [20]. The aetiology of the disorder is not established. Some experts believe that *Borrelia burgdorferi* (*Bb.*) can trigger an auto-immune response causing symptoms that remain after the acute infection phase. Other experts suggest that PTLDS is the result of a persistent, difficult-to-detect infection. Some clinicians hypothesize that the symptoms of PTLDS are unrelated to the patient’s LB [20]. A clear answer is difficult to obtain due to the lack of diagnostic tools that would allow for unequivocal confirmation of a cure. The Centers for Disease Control and Prevention currently recommends two-tiered testing to confirm a diagnosis of LB: a conventional enzyme-linked immunoassay (ELISA) test, followed by a Western blot test (WB) [21,22]. The first step uses a sensitive enzyme immunoassay or, rarely, an indirect immunofluorescence assay. If the test is borderline or positive, the sample is retested using separate immunoglobulin M (IgM) and immunoglobulin G (IgG) Western blots as the second step. The WB is interpreted using standardized criteria (2 of 3 signature bands for a positive IgM WB, and 5 of 10 signature bands for a positive IgG WB) [22]. The problems with this algorithm are its low sensitivity during early infection, subjective interpretation of the bands, and confusion among healthcare providers and patients regarding interpretation. This justifies the search for methods of diagnosing and monitoring the course of LB in different populations.

The aim of our study was to assess the dynamics of anti-VlsE (variable major protein-like sequence, expressed) IgG antibodies in children with the neurological manifestation of LB after antibiotic therapy.

## 2. Material and Methods

The prospective survey, based on the clinical observation of 40 children diagnosed with LB with neurological presentation: NB and FNP (ICD-A69.2) was conducted at the Department of Infectious Diseases and Paediatric Neurology Karol Jonscher Hospital University of Medical Sciences in Poznań) from 1 January 2019 to 30 December 2022.

We adopted the Polish Society of Epidemiologists and Infectiologists criteria as the basis for the diagnosis of LB [23]. They included the occurrence of clinical symptoms of the disease (in our study: neurological) and confirmation of *Bb.* infection by two-stage blood serological diagnostics. In the first stage, the ELISA test was performed, and following a positive result the second stage of the diagnostic, the WB test, was conducted. The inclusion criterion was a positive result for *Borrelia*-specific antibodies of classes IgG and IgM ELISA blood test confirmed by a WB blood test. We take all blood samples after patients’ admission to the ward, before antibiotic therapy. Serological tests of serum and cerebrospinal fluid (CSF) and analysis of intrathecal immunoglobulin concentration were performed in the Central Laboratory in H. Święcicki Clinical Hospital in Poznań. Quantification of specific antibodies to *Borrelia burgdorferi sl.* was conducted using the commercial kit (the CLIA method; Liaison system; DiaSorin SpA, Saluggia, Italy). Positive blood samples were confirmed by a WB test (EUROLINE Borrelia-RN-AT; Euroimmun AG, PerkinElmer, Inc., Waltham, MA, USA). Infections with pathogens, such as *Coxsackie*, *Herpes simplex* 1 and 2, Epstein–Barr, *Flaviviridae* viruses (mainly: tick-borne encephalitis), *Cytomegalovirus*, and *Mycoplasma pneumoniae*, were excluded in all NB patients using ELISA tests in Central Laboratory in Karol Jonscher Hospital and Voivodship State Sanitary—Epidemiological Station, both in Poznań.

All children were admitted to the hospital with neurological symptoms characteristic of the pathology (headache, vomiting, altered consciousness, and cranial nerve paralysis). Initially, all patients underwent paediatric and neurological examinations, which led to the suspicion of meningitis and/or facial palsy. The patients underwent a brain examination using computed tomography, in which we excluded the presence of focal processes and contraindications to lumbar puncture. We performed the computed tomography using a 128-layer Somatom Definition AS apparatus (Siemens). Subsequently, the patients underwent a lumbar puncture to obtain cerebrospinal fluid for further examination. Biochemical tests of CSF, such as the assessment of the number of leukocytes in 1 mL of liquid and analysis of protein and glucose concentration, were performed at the Central Laboratory of Karol Jonscher Hospital in Poznań, using the XN-1000 Sysmex analyser.

The diagnosis of Lyme meningitis was qualified by typical neurological symptomatology and confirmation of inflammatory changes in the patient’s CSF, including pleocytosis, defined as the level of leukocytes above 5 cells in 1 mL of CSF, positive results on ELISA and WB tests for LB made from the serum and CSF, and an increased intrathecal immunoglobulin concentration.

We defined the FNP as an acute facial nerve palsy involving the muscles in both the upper and lower parts of the face, either unilateral or bilateral. In patients with FNP, pleocytosis and intrathecal immunoglobulin synthesis were not detected in CSF. The ELISA and WB tests for *Bb.* were only positive for the blood, not for the CSF.

All patients received antibiotic treatment in accordance with Polish recommendations for the LB treatment with intravenous ceftriaxone for 21–28 days [23]. For registration reasons (contraindicated below 12 years of age), the use of oral doxycycline has been limited to only a few adolescents with isolated FNP.

At the time of discharge home, the children’s guardians received an invitation to continue the care and follow-up examinations at the Outpatient Clinic of Infectious Diseases.

The control group consisted of 36 patients hospitalized in the same ward with a diagnosis of aseptic meningitis and FNP in whom we excluded LB. The aetiologies of the observed disorders were *Coxsackie*, *Herpes simplex*, and Epstein–Barr virus infections. The children from the control group received the same clinical, laboratory, and imaging diagnostics as the patients from the study group. The inclusion criterion was negative for *Bb.* specific antibodies ELISA blood test with negative WB blood test.

The research was conducted with the consent of the Ethics Committee of Karol Marcinkowski University of Medical Sciences in Poznań (protocol code: 65/19; date of approval: 10 January 2019).

### 2.1. Quantitative Assessment

We conducted the study at the Department of Clinical Immunology, Medical University of Karola Marcinkowski in Poznań with the prior consent of the Head of the Department of Clinical Immunology, Professor Grzegorz Dworacki. For this study, serum was collected three times from patients in the study group and once from patients in the control group. The first collection took place after the diagnosis and before starting antibiotic therapy. The second collection (only from patients in the study group) took place 6 months after the first collection, i.e., after the end of treatment. The third collection of material for laboratory tests took place 12 months after the first collection.

Euroimmun’s Anti-Borrelia plus VIsE ELISA IgG Kit (EI 2132-9601-2 G) was used for the in vitro quantification of human IgG antibodies against *Bb.* antigens. Calibrators 1, 2, and 3, positive and negative controls, and test samples were used for quantification. Both positive and negative controls serve as internal controls for the reliability of the test procedure. The valid ranges of positive and negative controls are 80–148 RU/mL and 0–15 RU/mL, respectively.

The study material was human serum. Patient samples were diluted 1:101 in sample dilution buffer. According to the scheme, 100 µL of calibration serum, positive and negative control serum, and diluted patient sera were pipetted into the reaction wells, which were incubated for 30 min at room temperature. After 0.5 h, the reaction wells were emptied and washed three times, each time using 300 µL of wash buffer. The wash buffer remained in the well for 60 s each time, and then 100 µL of enzyme conjugate solution labelled with anti-human IgG peroxidase was pipetted onto each reaction well and the plates incubated for 30 min at room temperature. The wash solution was aspirated from the reaction wells and the washing procedure was repeated. After the washing step, 100 µL of the chromogen solution was pipetted into each reaction well. It incubated for 15 min at room temperature, protected from sunlight. To stop the procedure, 100 µL of the quenching solution was pipetted into each reaction well. A photometric evaluation of the colour intensity at a wavelength of 450 nm was performed. The following criteria were used to assess the results of laboratory tests: concentration of tested antibodies <16 RU/mL, negative; ≥16 to <22 RU/mL, borderline; and ≥22 RU/mL, positive.

### 2.2. Statistical Analysis

Statistical analyses were performed using the STATISTICA 10 PL statistical package. At all levels of the research and analysis, *p* < 0.05 was considered statistically significant.

## 3. Results

In the study group of 40 children with LB, the mean age was 11.0 ± 4.3 years. The mean age of the children in the control group was 10.2 ± 4.9 years. The Mann–Whitney U test showed no significant difference in the age level between the study and the control group (Table 1).

The study group included 16 (40%) girls and 24 (60%) boys, and the control group consisted of 10 (27.8%) girls and 26 (72.2%) boys. Pearson’s chi-square test showed no significant difference in the gender structure between the study group and the control group (Table 1).

### 3.1. Period I—Clinical Analysis

The study group, patients (n = 40) at the time of diagnosis presented 1 to 2 clinical forms of LB. Among all children with NB, meningitis was the most common diagnosis occurring in 19 (47.5%) patients. Isolated FNP affected (11) 27.5% of children. In 10 (25%) patients, FNP and aseptic meningitis occurred simultaneously.

**Figure 1:**
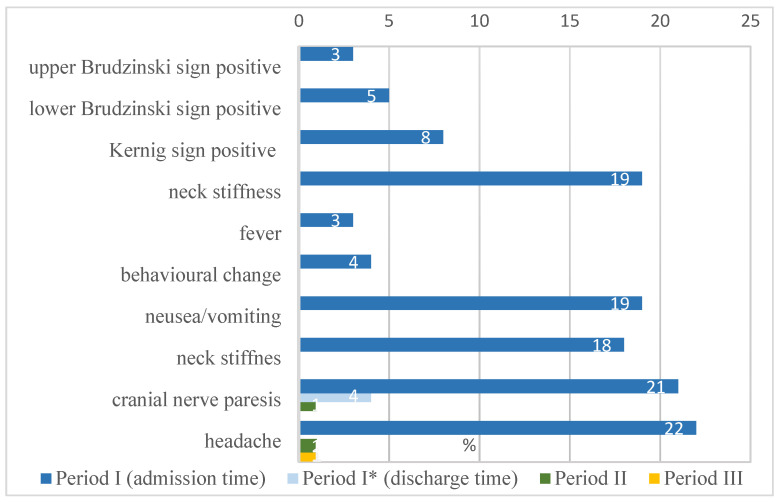
Clinical symptoms observed in NB children (n = 40) in the analysed periods.

The analysis showed headaches (22 patients), cranial nerve paresis (21 patients), nausea/vomiting (19 patients), and neck stiffness (19 patients) as the most common clinical symptoms observed in NB children in period I. In the majority of cases, the mentioned symptoms disappeared in period II. Headaches were reported by one patient and cranial nerve paresis by one patient. Only one patient reported headaches in the period III. The percentage distribution of abnormalities found in NB patients is shown in Figure 1.

### 3.2. Period I—Results of Anti-VlsE IgG Concentration Analysis

Regarding period I, defined as the moment before antibiotic therapy initiation, when the diagnosis of LB was made, the Pearson chi-square test showed a significant relationship in the evaluation of anti-VlsE IgG concentration between the study group and the control group (*p* < 0.0001). All patients in the study group had positive results, and all patients in the control group had negative results (Table 2).

### 3.3. Period II—Clinical Analysis

In period II, 6 months from the initiation of antibiotic therapy (from period I), two families declared persistent symptoms related to past NB. One child from the meningitis subgroup declared the persistence of recurrent headaches up to 2–3 times a week. The patient was re-hospitalized to verify symptoms. During hospitalization, the patient underwent neuroimaging using magnetic resonance imaging without visualizing abnormalities. The electroencephalography was adequate for the child’s age without signs of slowing down or paroxysmal discharges. We performed the control lumbar puncture and CSF examinations analogous to those performed in period I were conducted. CSF analysis did not confirm pleocytosis and an increased intrathecal immunoglobulin concentration.

One patient had features of a minor FNP. As it was established, the parents did not continue the recommended outpatient rehabilitation. They also did not perform the home treatment. The patient was referred under the care of a rehabilitation centre.

### 3.4. Period II—The Evaluation of the Anti-VlsE IgG Concentration

In the evaluation of the anti-VlsE IgG concentration (RU/mL), the NW chi-square test showed a significant relationship between the study group and the control group (*p* < 0.0001). A positive result was obtained by 12 (30.0%) of the patients in the study group, a borderline result by 8 (20.0%), and a negative result by 20 (50.0%) (Table 2).

### 3.5. Period III—Clinical Analysis

The physical examination of the patients, performed 12 months from the start of antibiotic therapy (from period I), showed no clinical signs of infection. None of the parents reported behavioural disorders analogous to those found in the period I. There was also no persistence of FNP or its recurrence.

Clinical analysis showed that only one patient (the same as period II) continued to experience headaches. They were sporadic, 1–2 times a week and did not interfere with the functioning of the child. The patient underwent clinical reassessment, which showed no neuroimaging abnormalities. Electroencephalography remained normal for the age. The psychological assessment indicated the possibility of the impact of a stressful home situation on the occurrence of headaches. The patient underwent further psychological care.

### 3.6. Period III—The Evaluation of the Anti-VlsE IgG Concentration

Only twenty-four patients from the study group reported for the study in period III. Some caregivers decided not to perform blood examination III due to the perfect health condition of the child and the lack of disturbing symptoms. In period III, after 12 months from the start of antibiotic therapy (from period I), the NW chi-square test showed a significant relationship in the evaluation of the anti-VlsE IgG concentration between the study group and the control group (*p* = 0.0206). In the study group, 83.3% of children and in the control group, 100% of people had a negative result. In the study group, one (4.2%) patient obtained the positive result, and three (12.5%) children had a borderline score (Table 2).

### 3.7. Results of Anti-VlsE IgG Concentration Analysis—Comparison of Periods I, II and III

The study of the concentration of VlsE antibodies (RU/mL) allowed us to demonstrate the statistical significance of differences between the control group and the study group for each measurement period using the calculations of the Mann–Whitney U test (*p* < 0.05). Higher values of VlsE concentration (RU/mL) in each measurement period were observed in children in the study group (mean value for control group—7.4 RU/mL; study group in period I—82.0 RU/mL (Z = 7.5); study group in period II—32.2 RU/mL (Z = 5.1); and study group in period III—12.7 RU/mL (Z = 2.6).

We used the Kruskal–Wallis test to examine the differences in the level of anti-VlsE IgG concentrations between periods in the study group. It showed a statistically significant difference (*p* < 0.0001). The mean and median concentrations of anti-VlsE IgG was highest in period I and lowest in period III. The multiple comparison test (post hoc test) showed statistically significant differences in the concentrations of the tested antibodies between measurement periods I and II (*p* < 0.0001) and I and III (*p* < 0.0001). There was no significant difference in the concentration of anti-VlsE IgG between periods II and III; however, the determined probability level (*p* = 0.0561) was close to the limit of significance. The concentration of anti-VlsE IgG in children in the study group decreased from one measurement period to the next (Table 3).

### 3.8. Diagnosis and Concentration of Anti-VlsE IgG

The study group was divided into three subgroups depending on the clinical diagnosis: FNP, NB, and a subgroup of patients with both disorders concurrently. Among the infected patients, meningitis was diagnosed in 29 (72.5%) children and facial nerve palsy in 21 (52.5%) children. Ten (25.0%) children had two diagnoses (FNP and meningitis). In the study group, the Kruskal–Wallis test showed no significant difference in the concentration of anti-VlsE IgG between patients with different diagnoses for any of the study periods (*p* > 0.05) (Table 4).

## 4. Discussion

Neuroborreliosis is a form of LB that affects various structures of the central and peripheral nervous system. The disease can be biphasic. In the early phase of NB, symptoms persist for up to 6 months, and in the late phase, symptoms occur from 6 months to several years after infection. In the early stages, paralysis of the cranial or peripheral nerves or/and meningitis, encephalitis, or encephalomyelitis are encountered. In the late period, NB may take the form of encephalomyelitis with a slow, progressive course with involvement of the white matter [14,15,19]. A relatively rare form of late NB is cerebral Borrelia vasculitis, which can lead to a cerebral infarction or stroke [24,25]. Late NB can occur as peripheral neuropathy characterized by sensory disturbances, paraesthesia, radicular pains, and sometimes paresis. The patients with PTLDS may report similar symptoms [19,20,26].

In children, more than 90% of NB cases have the form of subacute lymphocytic meningitis, FNP, or Banwarth syndrome, i.e., the coexistence of meningitis, cranial nerve palsy, and painful polyradiculoneuropathy [8]. Symptoms of Lyme meningitis often develop insidiously, and some patients and even clinicians, despite a positive response in the epidemiological interview (stay in an endemic area, presence of a tick bite, and even the occurrence of a typical skin lesion), do not connect them with LB. Meningitis is the manifestation observed in approximately 1% of children with LB [16]. The pathology is characterized by an afebrile and usually low-dynamic course, and the diagnosis is often delayed. The patients usually present with fatigue and headaches with varying intensity. Disturbances in the patient’s state of consciousness and motor skills are rare. Symptoms may resolve without treatment, but the mild signs often persist for years in untreated patients [19,27].

FNP is the most common presentation in America, while adult patients with early LB acquired in Europe (mostly associated with *B. garinii* infection) usually present with Bannwarth syndrome, a subacute meningoradiculoneuritis characterized by painful radiculitis and lymphocytic pleocytosis, which is sometimes accompanied by cranial nerve involvement [10,28]. Cranial nerve palsies, especially those of FNP, are also a manifestation of LB in children [29]. In a prospective cohort of 201 American children with LB, 3% of patients presented with FNP [26]. Our observations are consistent with those reported by authors from the Netherlands who confirmed the prevalence of FNP as 57% versus 43% for meningitis [30].

The diagnosis of NB is based on clinical and laboratory findings. In Poland, two-stage diagnostics based on antibody detection in the blood and CSF remains the standard [23]. This analysis is effective in diagnosis; however, it is not relevant for monitoring the treatment of a patient with neurological symptoms. No method in use could provide reliable information on the *Bb.* eradication from the child’s body and CSF [31].

Monitoring children who have had NB is not a procedure recommended by European infectious societies. The treatment used, mostly based on intravenous ceftriaxone, is considered highly effective. The evaluation criterion remains the clinical assessment of children, i.e., the disappearance of symptoms of FNP or positive meningeal symptoms, which is highly subjective, especially in the case of a patient with PTLDS. Performing multiple lumbar punctures to obtain CSF for monitoring the course of NB in a child raises ethical concerns as it is a traumatic procedure for the patient. In addition, currently used tests, including intrathecal production of antibodies and serological tests, are not the basis of cure assessment [12]. Published in 2005 by the Swiss Society of Infectious Diseases, a case definition for PTLSD does not differentiate between adults and children. Meanwhile, clinical observation indicates the presence of other stimulating factors in children, including the importance and dependence on decisions and interpretation of symptoms by the caregiver [32]. The search for diagnostic methods that would allow for the subjective diagnosis of children seems crucial and would allow for better cooperation with parents.

The quantitative assessment of the concentration of anti-VlsE antibodies of the IgG class is of interest to many authors [33,34]. The immunogenic VlsE antigen is a Bb-immunogenic 35-kDa outer surface lipoprotein. The VlsE protein contains conserved domains at the amino and carboxyl termini separated by a variable region. The fluctuating domain contains six variable regions (VR) and six invariable regions (IR). The six IR are interspersed in the variable domain and conserved among strains and genospecies of the *B. burgdorferi* sensu lato complex. During LB, the six VR routinely undergo sequence variation by recombination, a process recognized as a key mechanism of immunity. Of considerable significance is that the IR (particularly IR6) are also immunodominant [33]. Studies using sera from patients with LB have demonstrated an intense humoral response against VlsE in all stages of the disease, including the early stage of LB. The concentration of anti-VlsE antibodies is higher in people with advanced disease than in cases without residual LD symptoms after effective antibiotic therapy [33,34,35].

In our study, the comparison between the classic two-tiered procedure and the anti-Borrelia plus VIsE ELISA IgG KitQuick Test reported a high correlation between tests in untreated patients. The specificity of the VIsE ELISA IgG test was also confirmed (no positive results in patients in whom LB was excluded). The research hypothesis assumed that the concentration of the virus would decrease over time, and the quantitative assessment of antibodies in the blood allow for the monitoring of patients after NB treatment. It would be interesting to study whether the concentration would diminish to the values observed in previously uninfected people. The performed analysis confirmed that in cases with an early NB, the concentration of anti-VlsE IgG antibodies decreased significantly after 6 months of treatment and, in a considerable proportion (83.3%), reached values assumed as negative after 12 months. Similar dynamics were observed in children with NB, regardless of the type of neurological symptoms. The results of our study are consistent with the data presented by researchers from another Polish centre who assessed the dynamics of anti-VlsE antibodies in adult patients with early-phase LB with skin presentation: EM and MEM [31].

The scientific literature about lipoprotein anti-VlsE IgG antibodies allows for this antigen to be treated as a good marker of confirmed significance in the diagnosis of LB, so the assessment of antibody dynamics seems to be an effective tool for evaluating children after NB treatment [33]. This observation requires further analysis, including research on a much larger population. The limitation of our study was that it used a relatively small group of patients. We want to emphasize that the analysis was a pilot study. So far, no studies have been conducted on patients with NB, including in the paediatric population.

Based on our research and the available publications, the diagnostic key seems to be the multiple use of a quantitative assessment of the anti-VlsE IgG concentration in monitoring the efficacy of NB therapy through examination performed at three stages: first, at the time of introduction of antibiotic therapy; and later, 6 and 12 months after treatment [35,36]. The single assessment of anti-VlsE IgG concentration, as shown by other studies, does not seem to have practical significance. Determinations performed over time, in periods of at least 3–6 months, showing a significant decrease, correlating with the resolution of the child’s neurological symptoms, may be an additional diagnostic tool, which improves diagnosing PTLSD in paediatrics. The issue of PTLSD in children certainly requires further research on a larger population.

## Figures and Tables

**Table 1:** Age and gender analysis in the groups qualified for the study.

Group	n	Gender							Age (Years)					
		F/M	n	%	χ^2^	df	*p*	Mean	Stand. Deviation	Median	Min	Max	Z	*p*
Study	40.0	F	16.0	40.0	1.26	1.0	0.2621	11.0	4.3	11.0	4.0	17.0	0.54	0.5907
		M	24.0	60.0										
Control	36.0	F	10.0	27.8				10.2	4.9	9.0	1.0	17.0		
		M	26.0	72.2										

n—population size; χ^2^—Pearson chi-square test value; df—number of degrees of freedom; F-female; M-male; *p*—probability level; Z is the value of the Mann–Whitney U test; min.—minimum; max.—maximum.

**Table 2:** Assessment of the anti-VlsE IgG concentration score between the test and control groups in periods I, II, and III and the results of the chi-square test (period I—Pearson test; period II and III—NW test).

Period	Result	Group				χ^2^	df	*p*
		Study		Control				
		n	%	n	%			
I	Negative	0	0	36	100	76.0	1	<0.0001
	Positive	40	100	0	0			
	Total	40	100	36	100			
II	Negative	20	50.0	36	100.0	31.2	2	<0.0001
	Border	8	20.0	0	0.0			
	Positive	12	30.0	0	0.0			
	Total	40	100.0	36	100.0			
III	Negative	20	83.3	36	100.0	7.8	2	0.0206
	Border	3	12.5	0	0.0			
	Positive	1	4.2	0	0.0			
	Total	24	100.0	36	100.0			

χ^2^—Chi-square test value; df—number of degrees of freedom; *p*—probability level.

**Table 3:** Descriptive statistics of anti-VlsE IgG concentration (RU/mL) between the study and the control group and the results of the Mann–Whitney U test.

Group/Period	n	Anti-VlsE IgG Concentration (RU/mL)					Z	*p*
		Mean	Standard Deviation	Median	Min.	Max.		
Control	36	7.4	4.4	6.4	0.0	15.9	-	-
Study/I	40	82.0	53.7	68.7	23.1	229.8	7.5	<0.0001
Study/II	40	32.2	40.4	15.9	3.3	188.2	5.1	<0.0001
Study/III	24	12.7	10.1	11.9	1.7	53.4	2.6	0.0081

I—measurement period before the initiation of antibiotic therapy; II—measurement period after 6 months from period I; III—measurement period after 12 months from period I; Z is the value of the Mann-Whitney U test, *p*—probability level; min.—minimum; max.—maximum.

**Table 4:** Descriptive statistics of the concentration of anti-VlsE antibodies (RU/mL) between patients with different diagnoses in the study group in period I, II and III and the results of the Kruskal–Wallis test.

Period	Diagnosis	n	Anti-VlsE Antibody Concentration (RU/mL)					H	df	*p*
			Mean	Standard Deviation	Median	Min.	Max.			
I	FNP	11	72.1	37.6	64.6	32.6	174.6	2.77	2	0.2504
	Meningitis	19	77.7	61.0	60.4	23.1	229.8			
	FNP + Meningitis	10	100.9	53.8	100.7	37.3	200.7			
II	FNP	11	18.0	21.4	12.6	3.3	79.8	3.18	2	0.2037
	Meningitis	19	39.1	52.0	16.3	4.1	188.2			
	FNP + Meningitis	10	34.6	28.5	21.3	7.7	80.6			
III	FNP	4	8.9	6.8	7.9	1.7	18.0	0.81	2	0.6673
	Meningitis	13	14.5	12.6	12.4	3.2	53.4			
	FNP + Meningitis	7	11.5	5.7	11.6	5.0	21.5			

FNP—facial nerve paralysis; H—Kruskal-Wallis’s test value; df—number of degrees of freedom; *p*—probability level; min.—minimum; max.—maximum.

## Data Availability

The original contributions presented in the study are included in the article; further inquiries can be directed to the corresponding author.
